# Web-based follow-up tool (ePIPARI) of preterm infants—study protocol for feasibility and performance

**DOI:** 10.1186/s12887-023-04226-4

**Published:** 2023-08-23

**Authors:** Tiina Saarinen, Milla Ylijoki, Liisa Lehtonen, Petriina Munck, Suvi Stolt, Helena Lapinleimu, Päivi Rautava, Leena Haataja, Sirkku Setänen, Marika Leppänen, Mira Huhtala, Katriina Saarinen, Linda Grönroos, Riikka Korja, on behalf of the PIPARI Study group, Mikael Ekblad, Satu Ekblad, Eeva Ekholm, Annika Eurola, Laura Haveri, Minttu Helin, Milka Hirvonen, Jere Jaakkola, Eveliina Joensuu, Max Karukivi, Pentti Kero, Katri Lahti, Tuomo Lehtonen, Annika Lind, Jonna Maunu, Eeva Mäkilä, Laura Määttänen, Pekka Niemi, Anna Nyman, Riitta Parkkola, Liisi Ripatti, Susanna Salomäki, Virva Saunavaara, Matti Sillanpää, Päivi Tuomikoski, Karoliina Uusitalo

**Affiliations:** 1 Department of Paediatrics and Adolescent Medicine, Turku University Hospital and University of Turku, Turku, Finland; 2 Department of Paediatric Neurology, Turku University Hospital and University of Turku, Turku, Finland; 3 Department of Psychology and Logopedics, Faculty of Medicine, University of Helsinki, Helsinki, Finland; 4 Department of Public Health, University of Turku, Turku, Finland; 5 Department of Pediatric Neurology, Children’s Hospital and Pediatric Research Center, Helsinki University Hospital and University of Helsinki, Helsinki, Finland; 6 Department of Public Health and Psychiatry, and University of Turku, Turku, Finland; 7 Department of Psychology, and Speech Pathology, University of Turku, Turku, Finland

**Keywords:** eHealth, Neonatal follow-up, NICU, Neurodevelopment, Parental wellbeing, Early intervention

## Abstract

**Background:**

Preterm infants have a risk of health and developmental problems emerging after discharge. This indicates the need for a comprehensive follow-up to enable early identification of these problems. In this paper, we introduce a follow-up tool “ePIPARI – web-based follow-up for preterm infants”. Our future aim is to investigate whether ePIPARI is a feasible tool in the follow-up of preterm infants and whether it can identify children and parents in need of clinical interventions.

**Methods:**

ePIPARI includes eight assessment points (at term age and at 1, 2, 4, 8, 12, 18, and 24 months of corrected age) when the child´s health and growth, eating and feeding, neurodevelopment, and parental well-being are evaluated. ePIPARI consists of several widely used, standardized questionnaires, in addition to questions typically presented to parents in clinical follow-up visits. It also provides video guidance and written information about age-appropriate neurodevelopment for the parents.

Parents of children born before 34 weeks of gestation during years 2019–2022 are being invited to participate in the ePIPARI study, in which web-based follow-up with ePIPARI is compared to clinical follow-up. In addition, the parents of children born before 32 weeks of gestation, who reached the corrected age of two years during 2019–2021 were invited to participate for the assessment point of 24 months of ePIPARI. The parents are asked to fill in the online questionnaires two weeks prior to each clinical follow-up visit.

**Discussion:**

The web-based tool, ePIPARI, was developed to acquire a sensitive and specific tool to detect infants and parents in need of further support and clinical interventions. This tool could allow individualized adjustments of the frequency and content of the clinical visits.

**Trial registration:**

ClinicalTrials.cov, NCT05238168. Registered 11 April 2022 – Retrospectively registered.

## Background

Advances in perinatal and neonatal care have led to better survival of preterm infants from early gestational weeks [1, 2], which have led to increased need for neonatal follow-up services. Preterm birth is associated with problems in later growth, health, and neurodevelopment [3–6]. Parents of preterm infants also have an increased risk for stress, anxiety, and depression [7–9] that may negatively affect their child’s well-being and development throughout childhood. Literature suggests that especially very preterm (< 32 weeks’ gestation) infants should have careful follow-up for early identification of prematurity-related problems [10, 11]. However, also infants born moderate and late preterm (33 − 36 weeks of gestation) have shown to be in a higher risk of long-term health and neurodevelopmental sequelae compared with infants born at term [12, 13].

The European Foundation for the Care of Newborn Infants has recently recommended the follow-up of very preterm children with standardized assessments of neurodevelopment and behavior at 2 years of age and at transition to school [14]. Targeted screening of parental mental health through regular follow-up visits is also included in recommendation [14]. The Swedish national recommendation includes follow-ups at 2 and 5.5 years of age for extremely preterm infants born below 28 weeks of gestation and for all preterm infants with brain injuries, severe morbidity, or severe growth retardation [15]. The National Institute for Health and Care Excellence guidelines for the United Kingdom recommend a two-year follow-up for all children born below 30 weeks of gestation. In addition, children born between 30 and 36 weeks of gestation should be followed if they have additional risk factors of abnormal development [16]. Although the importance of neonatal follow-up is internationally recognized, there is a need for structured evidence based multidisciplinary follow up practices. It is important to develop evidence-based follow-up programs to improve cost-efficiently the quality of the follow-up of preterm infants and to create opportunities for a more uniform follow-up practice. Systematic follow-up and timely interventions make it possible to target the limited resources and the services effectively to those children and families who need them most.

eHealth Screening and Support tools have been suggested as being useful in early intervention and personalised support [17, 18] and could facilitate the follow-up programs of preterm infants with risks. Internet-based interventions and digital materials have shown to be beneficial in paediatric mental health problems and in parental support [19–22]. The use of a range of approaches for example in intervention and supporting families is also recommended in national recommendations for premature infants [14, 16]. As there are no previous reports on web-based follow-up tools for preterm infants that cover all aspects of developmental follow-up, that we are aware of, ePIPARI- a follow-up tool for preterm children was developed in Turku University Hospital based on clinical knowledge and relevant international literature as well as expertise gained from the PIPARI-study (The Development and Functioning of Very Low Birth Weight Infants from Infancy to School Age) (www.utu.fi/pipari).

Our aim was to develop a web-based tool that could supplement the follow-up and family intervention services of preterm infants so that different needs and health care resources could be met sufficiently. The goal of web-based tool, ePIPARI, is to target the resources and the services cost- effectively to those preterm children and families who need them most, and also enable the follow-up of low risk late preterm infants otherwise left out of the clinical follow-up due to limited resources. The purpose is to enhance the follow-up through web-based monitoring; reduce the amount of unnecessary routine visits for very preterm children doing well and also identify those in need for additional visits and interventions. Also, by reducing the amount of unnecessary routine visits, digital follow-up could also be offered for all late preterm infants and according to the answers in the ePIPARI, those children and families in need can be called for clinical visits. For the clinicians, ePIPARI provides an additional tool to obtain information e.g. about parental well-being and the child’s socio-emotional development indicating increased risk for later psychiatric symptoms. Previous studies suggest that it is easier for clients and parents to report their sensitive information without face-to-face contact [17, 18]. Obtaining this information allows early intervention and further guidance.

In this paper, we introduce our web-based follow-up tool ePIPARI and the study protocol for evaluation. To evaluate the feasibility and performance of ePIPARI we designed a prospective study in which web-based follow-up with ePIPARI is compared to clinical follow-up.

## Methods

### Aims

The aim of this prospective study is to investigate whether the ePIPARI is a feasible and valid tool for follow-up of preterm infants in identifying children and families in need of further clinical interventions or support compared to clinical follow-up. We hypothesize that 1) ePIPARI is a feasible tool for the follow-up of preterm infants and their parents, and 2) it is comparable to routine clinical follow-up in recognizing children and parents in need for requiring interventions.

### The structure of ePIPARI

ePIPARI is a digital follow-up tool on a web-based platform. It provides comprehensive follow-up for preterm children and their parents from term age until 2 years of corrected age. ePIPARI-follow-up tool was developed by the multidisciplinary PIPARI Study group and the staff of the Pediatric Unit of Turku University Hospital and is based on international recommendations and relevant literature. In addition, clinical knowledge and expertise gained from the PIPARI-study have been utilized in the development of the tool. The PIPARI Study group consist of researchers and experts from different disciplines, including pediatric and adolescent medicine, nursing science, pediatric neurology, psychology, neuropsychology, speech-language pathology, public health, psychiatry, physiotherapy and neuroradiology. The content and questionnaires of each time point in ePIPARI were developed and planned in collaboration with this multidisciplinary team. The permission to use standardized assessments and copyright-related issues has been taken into account. In addition, the nurses of Preterm Infant Follow-Up Clinic and Neonatal Intensive Care of Unit participated in planning the content. The technical implementation of the web-based material was carried out by Kaiku Health-company, whose web-based platform was already in use at Turku University Hospital in Pediatric Oncology Unit.

ePIPARI includes eight assessment points: term age, 1, 2, 4, 8, 12, 18 and 24 months of corrected age. It evaluates the child´s health and growth, eating and feeding, neurodevelopment (development of motor skills, language, cognition, behavior, and socials skills) and family wellbeing (parental depression and marital satisfaction) (Table 1). Responses are classified for typical development, growth and behavior or responses of concern using different thresholds or a traffic light model. Widely used standardized questionnaires, and age-appropriate clinical questions are used (Table 2). ePIPARI also provides videos of health and typical development, as well as written information on how to support a child’s development and how to get help with concerns related to parenting or child’s developmental problems.

**Table 1:** ePIPARI – Web-based follow-up of preterm infants. Age points (corrected age) and topics

	Term age	1 month	2 months	4 months	8 months	12 months	18 months	24 months
Health	x	x	x	x	x	x	x	x
Growth	x	x	x	x	x	x	x	x
Feeding and eating	x	x	x	x	x	x	x	x
Motor development			x	x	x	x	x	x
Language development						x	(x)	x
Cognitive development								x
Behavior and self regulation							x^a^	x
Social relationships							x^a^	x
Interaction and attachment				x	x			x
Parental well-being, depression and marital satisfaction					x^a^	x^a^		

^a^questionnaires for both parents separately

(x) for children scored below the 10^th^ percentile value of the norming sample of the FinCDI-SF or FinCSBS-ITC at 12 months of corrected age

**Table 2:** ePIPARI—Web-based follow-up of preterm infants. Assessment methods

Language development	Finnish version of the Infant–Toddler Checklist of the Communication and Symbolic Behavior Scales Development Profile (FinCSBS-ITC) The Finnish Short Version of the MacArthur Communicative Developmental Inventories (FinCDI-SF)
Cognitive development	Parent Report of Children’s Abilities-Revised for preterm infants (PARCA-R)
Behavior and Self regulation	Brief Infant–Toddler Social and Emotional Assessment (BITSEA)
Social relationships	Brief Infant–Toddler Social and Emotional Assessment (BITSEA)
Parental well-being and functioning	Edinburgh Postnatal Depression Scale (EPDS) Revised Dyadic Adjustment Scales (RDAS)

#### Measures of the web-based follow-up

##### Health and growth

At every age-point there are general questions of health, growth, nutrition (type of milk, dietary supplements, solid food, vitamins), eating difficulties and medications, bowel function, skincare, vision and hearing and sleeping. Growth is evaluated by requesting the growth measures (weight, length, head circumference) from the last visit to the public well-baby clinic. Growth is evaluated using the Finnish age and sex specific growth charts and the growth screening program. General health/illness is evaluated by questions on any possible underlying illnesses, acute and previous infections, and different symptoms, for example, difficulties in breathing, posseting, constipation, and crying. Clinical problems are evaluated with categories of concern. Information on the sleeping habits of the child is also asked at each age point. A Patient and Public Involvement approach is included by asking the parents questions about concerns related to their child and opinions on the survey; “What would you like us to ask?” and “Did you find these questions relevant?”.

#### Neurodevelopment

*Motor development* is outlined with questions related to age-appropriate motor development at each age point beginning from 2 months of corrected age. Parents are asked for example if the child is able to maintain her/his head upright, whether the child uses both hands and legs equally, and what kind of postures and motility is typical for the child. The tutorial videos on ePIPARI are offered to help parents to answer the questions about motor skills. If there is any deviation from the expected age-appropriate level, additional detailed follow-up questions then follow. Concerns about motor development are evaluated using a traffic light model for each item, where green indicates that child is developing typically, yellow means a possible risk, and red a clear risk of developmental problems.

Information on the emerging *language and communication ability* is collected at 12 and at 24 months of corrected age using the Finnish short form versions of MacArthur Communicative Development Inventories, FinCDI-SF [23–25] and different composites of the Finnish version of the Infant–Toddler Checklist of the Communication and Symbolic Behavior Scales Development Profile (FinCSBS-ITC) [26, 27]. Both methods have been adjusted using norming and validated for the Finnish population, and they provide information on early receptive and expressive lexical development and early communicative behavior (i.e. expression of emotions, use of gaze, communication ability, gesture, and object use). The cut-off points of the norming population for these methods (the weakest 10^th^ percentile) are used to identify children at risk for language development and communication.

*Cognitive development is* assessed at 2 years of corrected age using a Finnish translation of the original English Parent Report of Children’s Abilities-Revised questionnaire (PARCA-R) validated for premature children [28, 29]. The original PARCA-R is a parent questionnaire concerning children’s nonverbal cognitive and language development at 2 years of age. It has been shown to be sensitive and specific in identifying a neurodevelopmental delay in children born preterm [29, 30]. In this protocol only the section assessing nonverbal cognition consisting of 34 items is used. The items are being summed to give a nonverbal cognitive subscale score. The cut-off point of 24 points (the weakest 10^th^ percentile) is used to identify children with developmental delay. The original PARCA-R version also includes a short form version of the Communicative Development Inventories in the language in question, and some items assessing language structures. In ePIPARI, the Finnish short form versions of MacArthur Communicative Development Inventories (FinCDI- SF) is used to collect information on lexical ability at two years of age. This information can be used together with nonverbal cognitive subscale.

*Behavior and social skills* are evaluated at 24 months of corrected age using the Brief Infant–Toddler Social and Emotional Assessment (BITSEA)—questionnaire including 42 items [31]. Based on the items, five subscales are formed; *externalizing subscale* e.g. aggression, overactivity, *internalizing subscal*e e.g. shyness, anxiety, *dysregulation problems subscale e.g*. sleeping, eating, and emotion regulation problems and *maladaptive behaviors subscales* e.g. behaviors related to autism spectrum disorder and *social competence* e.g. prosocial peer relations, empathy, play skills and social relatedness. The questions and interpretation of the results are separated into problem and competence categories. The first four subscales are combined to form a total problems score. The problem total cut-off score is set at the 25^th^ percentile, and the competence total cut-off score at the 15^th^ percentile and these are used to recognize the child’s risk of behavioral and social problems.

#### Family well-being

*Parental sleep quality* is evaluated at each age point, but a more detailed questionnaire is sent to both parents when the child is at 8 months of corrected age. The quality of parents’ sleep at 8 months of corrected age is assessed using 8 items extracted from the original Basic Nordic Sleep Questionnaire (BNSQ) [32, 33]. The *family`s well-being* is examined by asking about the social support they have received as regards formal or/and informal support, and the help they would like to have to give them more support with everyday life. *Parental depressive symptoms* are assessed separately from both parents when the child is at 8 months and 12 months of corrected age by *the Edinburg Postnatal Depression Scale* [34] which has been widely used to assess maternal depression. A cut-off value of 13 is used. In addition, *marital satisfaction* is assessed with The Revised Dyadic Adjustment Scale, RDAS [35] when the child is at 8 months of corrected age. The RDAS is a shortened version of the Dyadic Adjustment Scale [36]. It consists of 14 items designed to measure adjustment in dyadic relationships on 3 sub-scales: Consensus, Satisfaction and Cohesion.

#### Parent’s user-experience

To study the usability, parents are asked to anonymously complete enquiries about user-experience of the web-based follow- up via email. These enquiries ask parents if it has been very easy, easy, difficult, or very difficult to answer the questions, how time consuming they have found filling in the forms, whether the material provided by the service has been useful, and if they have experienced any benefits. Parents are also asked to give general feedback and improvement suggestions. The ePIPARI follow-up protocol can be improved during the study based on the experiences of the families or researchers.

### Information of routine follow-up as comparison

The routine follow-up of preterm infants in Turku University hospital serves as a comparison for the performance of ePIPARI. The children born < 32 weeks or ≤ 1500 g are routinely followed systematically until the corrected age of two years, while the infants born between 32 and 34 weeks of gestation (and ≤ 1500 g) participate in routine follow-up visits at least at 2, 4 and 8 months of corrected age and the follow-up continues until they are able to stand up independently (usually until 1 year of corrected age). The follow-up focuses on identifying issues that require clinical evaluation, such as concerns about growth and feeding, neurodevelopment, and parental wellbeing. The structure of the routine follow-up is presented in Table 3.

**Table 3:** Clinical follow-up schedule for very preterm infants at the University Hospital of Turku, Finland

Assessment methods and examinations	Term age	1 month	2^a^ months	4^a^ months	8 months	12^a^ months	24 months
Clinical examination by pediatrician and physiotherapist	x	x	x	x	x	x	x
Growth: weight, length, and head circumference by registered nurse	x	x	x	x	x	x	x
Dubowitz neurological examination by physiotherapist	x	x	x	x			
Hammersmith Infant Neurological Examination by physiotherapist						x	
Finnish Long Form Version of the MacArthur Communicative Developmental Inventories (questionnaire)						x	
Bayley Scales III (Infant and Toddler Development) by psychologist and physiotherapist							x
Edinburgh Postnatal Depression Scale (EPDS) for both parents (questionnaire)				x			
Magnetic Resonance Imaging of the brain	x						
Brainstem Auditory Evoked Potential		x					

In addition to the schedule above, the follow up includes ophthalmological examination at 3–3,5 years of age. All ages are corrected for prematurity

^a^for infants born at gestational weeks 32–34 GA

### Participants and recruitment procedure

The recruitment process is ongoing. Parents of all preterm children, who meet the inclusion criteria during 4/2019–12/2022 are approached before discharge and asked to participate in the “ePIPARI—web-based follow-up of preterm infants”. The inclusion criteria for the infants and the parents are 1) infant is born before 34 gestational weeks, 2) infants have systematic developmental follow-up in pediatric unit in Turku University Hospital and 3) parents speak Finnish, Swedish or English. Inclusion criteria for participating only in the two-year corrected age was that the child is born before 32 gestational weeks during 2019–2021. We excluded infants, who didn’t have clinical follow-up in Turku University Hospital and parents, who couldn`t speak Finnish, Swedish or English.

Recruitment takes place in the neonatal intensive care unit by a registered nurse or a PhD-student (T.S) and the link for registration is sent to the parents’ email. After registration, parents log into ePIPARI via email or via a Mobile App by using a secure strong recognition. Advice and support for logging in is provided by a nurse before discharge. All preterm born children have the routine follow-ups regardless of their participation in the study.

The ongoing data collecting process is as follows: A digital invitation to complete the age-point specific questionnaires is sent two weeks prior to each age-point to parents’ email or to Mobile App. Parents can complete the questionnaires together or only by another, but queries related to family well-being are sent to both parents separately (Table 1). Reminders are sent if the questionnaires are not filled in. The aim is for the questionnaires to be completed before the clinical follow-up visits and so that afterwards the answers can be compared to the (written) medical records made in the clinical visits. The staff of the Pediatric unit will be blinded for the data of the ePIPARI so that it does not affect their assessment during clinical visits. Experienced medical doctors evaluate the answers of the questionnaires at two weeks intervals and estimate the amount of concern based on the responses and pre-set cut-off points. If there is significant concern that was not evident during the clinical follow-up visit, the medical doctor contacts the family or staff of the neonatal follow-up clinic.

### The evaluation of feasibility and performance

The evaluation of feasibility is based on the knowledge of how parents have taken part in the study and how they have completed the questionnaires of ePIPARI at different age points. Furthermore, the anonymous survey of user-experience provides descriptive information on how parents have experienced the questionnaire, the technical usability of the ePIPARI, and its content.

The validity of the ePIPARI is evaluated based on its ability to identify children and parents in need of further support and clinical interventions in comparison to clinical visits. The outcomes to be compared are: concerns requiring clinical intervention including specialist consultations, further investigations, additional controls/follow-up visits, prescription medication or supplements or referral for treatment or therapy.

### Sample size and statistical power

Approximately 50–60 very preterm infants enter the clinical follow-up at Turku University Hospital every year and 600–650 children born prematurely are examined in the Preterm Infant Follow-up Clinic each year. Power calculations were performed to detect the children needing interventions or further follow up at 2 years of corrected age. Using data collected from ePIPARI for this age-point, we get the minimum sample size 26 participants, to achieve a power of 80% and a two-sided significance of 5% for detecting a difference of 35% between web-based and clinical evaluation (42% vs 77%) for further follow-up need. While both evaluations are made for all children, also moderate association between evaluations was assumed (correlation between paired observations was assumed to be 0.35) [Reference: Dhand, N. K., & Khatkar, M. S. (2014). Statulator: An online statistical calculator. Sample Size Calculator for Comparing Two Paired Proportions. Accessed 25 April 2023 at http://statulator.com/SampleSize/ss2PP.html]. The estimated data collection time to achieve the predetermined number of participants is 2–3 years, as not all the families agree to participate in the ePIPARI Study. The data collection is ongoing, and the analysis will start when data has been accumulated.

### Plan for statistical analysis

Data will be presented using descriptive statistics: mean values (SD) for normally distributed data, median (range/min, max) for data that are not normally distributed, and counts with percentages for categorical data.

The main comparison between digital assessment and clinical evaluation for each timepoint will be done with McNemar test (binary categorization) or Bowker test (more than two categories) for disagreement. Digital assessment will be categorized with different thresholds or a traffic light model (“child is developing typically”, “a possible risk or a clear risk”) and clinical assessment will be categorized to categories: “no concern”, “concerns requiring clinical intervention including specialist consultations, further investigations, additional controls/follow-up visits, prescription medication or supplements or referral for treatment or therapy”. Also, agreement and it’s 95% confidence interval will be calculated. Furthermore, sensitivity, specificity, positive and negative predictive values will be calculated (compared to clinical assessment).

Pearson’s or Spearman’s correlation coefficients will be generated to evaluate the linear relation between continuous variables.

In addition, to study the association between predictor and response variables within ePipari or clinical assessment visits, linear models or logistic regression models can be built up. The effect of confounding factors (such as socio-economic status, pregnancy week when child was born) will be taken into account, as appropriate. For those measurements which are evaluate repeatable, linear mixed models suitable for repeated measurements will be used.

Statistical analyses will be performed using SPSS version 29 or later or SAS System, version 9.4 for Windows or later. Two-sided p-value < 0.05 will be considered as statistically significant. Confidence intervals of 95% will be calculated, especially for feasibility part of the study.

## Discussion

This paper describes the study protocol of the web-based follow-up tool ePIPARI for preterm infants using routine clinical follow-up visits in Turku University Hospital, Finland, as comparison. Evidence based and cost-efficient follow-up systems for preterm infants are needed to improve early identification of problems related to prematurity, to enable timely intervention and support for preterm infants and families, to monitor the quality of neonatal care and follow-up, and harmonize practices. Our systematic and intensive evidence-based follow-up enables the development of such a follow-up protocol.

The purpose of this ePIPARI-project is to create more individualized follow-up through web-based monitoring for very preterm infants; reduce the number of routine visits for those who grow and develop well without compromising their safety, and on the other hand provide targeted, time-specified, and more intensive care and support for those in need. ePIPARI could also enable follow-up and support for late-preterm infants for whom systematic follow-up in special health care services cannot be generally provided due to limited staff resources. We expect that digital monitoring will provide additional benefits to families; ePIPARI is a channel for dissemination information and support and may support a flexible follow-up tailored to the needs of the family.

The possible limitation of the study is the challenge of involving parents in a parallel follow-up. Even the use of routine follow-up services has been shown to be a challenge and less used especially among socially disadvantaged families [37, 38], who might benefit most from the follow up. Moreover, this web-based follow-up does not currently reach the immigrant families with no mutual language; however, these families may benefit more from clinical visits with an interpreter. However, we assume, that this web-based follow-up system will increase the involvement of the parents due to easy accessibility and availability.

In the future, this web-based system is intended to be shared with a larger number of NICUs and follow up services to increase comparison and cooperation and to enable children and parents to obtain support and help when needed. The long-term effects and the usability of the ePIPARI follow-up tool will be evaluated in this prospective study.

## Data Availability

We follow the confidentiality rules in terms of data sharing in the medical faculty at the University of Turku. There are no linked research data sets for this paper. Adhering to the EU General Data Protection Regulation (GDPR) and Finnish legislation concerning sensitive data such as health-related information, the authors are not authorized to share the data without proper permission to conduct new research.

## Abbreviations

BITSEA: Brief Infant–Toddler Social and Emotional Assessment
BNSQ: Basic Nordic Sleep Questionnaire
DAS: Dyadic Adjustment Scale
EPDS: Edinburgh Postnatal Depression Scale (EPDS) for both parents
FinCDI-SF: Finnish short form versions of MacArthur Communicative Development Inventories
FinCSBS-ITC: Finnish version of the Infant–Toddler Checklist of the Communication and Symbolic Behavior Scales Development Profile
PARCA R: Parent Report of Children’s Abilities-Revised for preterm infants
RDAS: Revised Dyadic Adjustment Scales

## References

[1] Venkatesh KK, Lynch CD, Costantine MM, Backes CH, Slaughter JL, Frey HA (2022). Trends in active treatment of live-born neonates between 22 weeks 0 days and 25 weeks 6 days by gestational age and maternal race and ethnicity in the US, 2014 to 2020. JAMA.

[2] Groenendaal F, Termote JU, van der Heide-Jalving M, van Haastert IC, de Vries LS (2010). Complications affecting preterm neonates from 1991 to 2006: what have we gained?. Acta Paediatr.

[3] Power VA, Spittle AJ, Lee KJ, Anderson PJ, Thompson DK, Doyle LW, Cheong JLY (2019). Nutrition, growth, brain volume, and neurodevelopment in very preterm children. J Pediatr..

[4] Saigal S, Doyle LW (2008). An overview of mortality and sequelae of preterm birth from infancy to adulthood. Lancet.

[5] Broström L, Vollmer B, Bolk J, Eklöf E, Ådén U (2018). Minor neurological dysfunction and associations with motor function, general cognitive abilities, and behaviour in children born extremely preterm. Dev Med Child Neurol.

[6] Setänen S, Lahti K, Lehtonen L, Parkkola R, Maunu J, Saarinen K, Haataja L (2014). Neurological examination combined with brain MRI or cranial US improves prediction of neurological outcome in preterm infants. Early Hum Dev.

[7] Korja R, Savonlahti E, Ahlqvist-Bjorkroth S, Stolt S, Haataja L, Lapinleimu H (2008). Maternal depression is associated with mother-infant interaction in preterm infants. Acta Paediatr.

[8] Huhtala M, Korja R, Lehtonen L, Haataja L, Lapinleimu H, Rautava P, PIPARI Study Group (2014). Associations between parental psychological well-being and socio-emotional development in 5-year-old preterm children. Early Hum Dev.

[9] Huhtala M, Korja R, Lehtonen L, Haataja L, Lapinleimu H, Munck P, Rautava P, the PIPARI Study Group (2012). Parental psychological well-being and behavioural outcome of very low birth weight infants at 3 years. Peadiatrics.

[10] Pascal A, Govaert P, Oostra A, Naulaers G, Ortibus E, Van den Broeck C (2018). Neurodevelopmental outcome in very preterm and very-low-birthweight infants born over the past decade: a meta-analytic review. Dev Med Child Neurol.

[11] Leppänen M, Pape B, Ripatti L, Karukivi M, Haataja L, Rautava P. Burden of mental, behavioral, and neurodevelopmental disorders in the Finnish most preterm children: a national register study. Eur Child Adolesc Psychiatry. 2023. 10.1007/s00787-023-02172-1. Epub ahead of print. PMID: 36847865. 10.1007/s00787-023-02172-1 PMC10869390 36847865

[12] Cheong JL, Doyle LW, Burnett AC (2017). Association between moderate and late preterm birth and neurodevelopment and social-emotional development at age 2 years. JAMA Pediatr.

[13] Sharma D, Padmavathi IV, Tabatabaii SA, Farahbakhsh N (2021). Late preterm: a new high risk group in neonatology. J Matern Fetal Neonatal Med.

[14] Follow-up and continuing care (2018). München: European Foundation for the Care of Newborn Infants (ECFNI).

[15] Svenska Neonatalföreningen (2015). Nationella riktlinjer för uppföljning av neonatala riskbarn.

[16] National Guideline Alliance (UK). Developmental follow-up of children and young people born preterm. London: National Institute for Health and Care Excellence (NICE); 2017. 28837304

[17] Lingely-Pottie P, McGrath PJ (2006). A therapeutic alliance can exist without face-to-face contact. J Telemed Telecare.

[18] Lingley-Pottie P, McGrath PJ (2008). A paediatric therapeutic alliance occurs with distance intervention. J Telemed Telecare.

[19] Sourander A, McGrath PJ, Ristkari T, Cunningham C, Huttunen J, Lingley-Pottie P (2016). Internet-assisted parent training intervention for disruptive behavior in 4-year-old children: a randomized clinical trial. JAMA Psychiat.

[20] McGrath PJ, Lingley-Pottie P, Thurston C, MacLean C, Cunningham C, Waschbusch DA (2011). Telephone-based mental health interventions for child disruptive behavior or anxiety disorders: randomized trials and overall analysis. J Am Acad Child Adolesc Psychiatry.

[21] Melnyk BN, Crean HF, Feinstein NF, Fairbanks E (2008). Maternal anxiety and depression after a premature infant's discharge from the neonatal intensive care unit: explanatory effects of the creating opportunities for parent empowerment program. Nurs Res.

[22] Feng YY, Korale-Liyanage S, Jarde A, McDonald SD (2021). Psychological or educational eHealth interventions on depression, anxiety or stress following preterm birth: a systematic review. J Reprod Infant Psychol.

[23] Stolt S, Vehkavuori S (2018). SANASEULA – MacArthur Communicative Development Inventories –arviointimenetelmän lyhyt, suomalainen versio. [The Finnish short form versions of the MacArthur Communicative Development Inventories].

[24] Fenson L, Pethic S, Renda C, Cox JL, Dale PS, Reznick JS (2000). Short-form versions of the Macarthur communicative development inventories. Appl Psycholinguist.

[25] Fenson L, Marchman VA, Thal DJ, Dale PS, Reznick JS, Bates E (2007). MacArthur-Bates Communicative Development Inventories User’s guide and technical manual.

[26] Laakso M-L, Eklund K, Poikkeus A-M (2011). Esikko. Lapsen esikielellisen kommunikaation ja kielen ensikartoitus.

[27] Wetherby AM, Prizant BM (2002). Communication and Symbolic Behavior Scales Developmental Profile. Infant-Toddler Checklist.

[28] Johnson S, Bountziouka V, Linsell L, Brocklehurst P, Marlow N, Wolke D, Manktelow B (2019). Parent Report of Children’s Abilities – Revised (PARCA-R).

[29] Johnson S, Marlow N, Wolke D, Davidson L, Marston L, O'Hare A (2004). Validation of a parent report measure of cognitive development in very preterm infants. Dev Med Child Neurol.

[30] Blaggan S, Guy A, Boyle EM, Spata E, Manktelow BN, Wolke D, Johnson S (2014). A parent questionnaire for developmental screening in infants born late and moderately preterm. Pediatrics.

[31] Briggs-Gowan MJ, Carter AS. Manual for the brief infant-toddler social and emotional assessment (BITSEA). San Antonio: Psychological Corporation; 2006.

[32] Partinen M, Gislason T (1995). Basic Nordic Sleep Questionnaire (BNSQ): a quantitated measure of subjective sleep complaints. J Sleep Res.

[33] Polo-Kantola P, Aukia L, Karlsson H, Karlsson L, Paavonen EJ (2017). Sleep quality during pregnancy: associations with depressive and anxiety symptoms. Acta Obsteticia et Gynecologica Scandinavica.

[34] Cox JL, Holden JM, Sagovsky R (1987). Detection of postnatal depression. Development of the 10-item Edinburgh postnatal depression scale. Br J Psychiatry.

[35] Busby DM, Crane DR, Larson JH, Christensen C (1995). A revision of the Dyadic Adjustment Scale for use with distressed and nondistressed couples: Construct hierarchy and multidimensional scales. J Marital Fam Ther.

[36] Spanier GB (1976). Measuring dyadic adjustment: New scales for assessing the quality of marriage and similar dyads. J Marriage Fam.

[37] Hintz SR, Gould JB, Bennett MV, Gray EE, Kagawa KJ, Schulman J, Murphy B, Villarin-Duenas G, Lee HC (2015). Referral of very low birth weight infants to high-risk follow-up at neonatal intensive care unit discharge varies widely across California. J Pediatr.

[38] Seppänen AV, Draper ES, Petrou S, Barros H, Andronis L, Kim SW, SHIPS Research Group (2022). Follow-up after very preterm birth in Europe. Arch Dis Child Fetal Neonatal Ed.

